# Modeling the Role of Epitaxial Grain Structure of the Prior β Phase and Associated Fiber Texture on the Strength Characteristics of Ti-6Al-4V Produced via Additive Manufacturing

**DOI:** 10.3390/ma13102308

**Published:** 2020-05-17

**Authors:** Michael D. Sangid, Andrea Nicolas, Kartik Kapoor, Eric Fodran, John Madsen

**Affiliations:** 1 School of Aeronautics and Astronautics, Purdue University, West Lafayette, IN 47907, USA; anicolas@purdue.edu (A.N.); kapoor0@purdue.edu (K.K.); 2 Northrop Grumman Aerospace Systems, El Segundo, CA 90278, USA; eric.fodran@ngc.com (E.F.); john.madsen@ngc.com (J.M.)

**Keywords:** titanium alloys, electron beam melting, microstructure characterization, crystal plasticity modeling, elasto-viscoplastic fast Fourier transform modeling, Burgers orientation relationship

## Abstract

Due to the rapid cooling and directional heat flow inherent in metal-based additive manufacturing, Ti-6Al-4V results in epitaxial grain growth and a fiber texture of the prior β phase. While Ti-6Al-4V produced via powder bed, electron beam melted processing can exhibit a range of strength characteristics, recent studies have shown superior strength properties, compared to similar orientations, of conventional plate material (AMS 4911) across a range of elevated temperatures (204 to 371 °C). To investigate this phenomenon, a series of crystal plasticity models was developed for the representative grain structures of Ti-6Al-4V to rationalize if the columnar, fiber texture produced by additive manufacturing (AM) was sufficient to explain the observed strength attributes. As a first step towards understanding this behavior, the grain structure was characterized via electron backscattering diffraction for AM material taken from four specimens (with different build directions), as well as material taken from baseline plate material (along and transverse to the rolling direction), and the resulting microstructures were modeled via a crystal plasticity framework. As expected, the results showed the AM material accounting for only the α grain structure was stronger in the vertical builds and weaker in the horizontal builds compared to the conventional plate counterparts. This suggested that grain morphology and α grain orientation alone provided some information about the relative strengths, but did not explain the overall trends observed from the experiments. To account for the role of texture, the characterized α phase was converted, via variant selection, to its prior β phase for use in the simulations. The results showed that each simulation of the AM prior β phase exhibited a higher strength compared to the baseline plate material, except for one specimen (horizontally built), which had large colonies of soft microtextured regions for the prior β structure. This suggests that some variability was experienced (as anticipated), but the texture (especially of the prior β macrozones) was a key contributor for the unusually high strength observed of the AM Ti-6Al-4V material.

## 1. Introduction 

Ti-6Al-4V is a dual (α + β) phase alloy that exhibits high strength, fracture toughness, fatigue performance, and resistance to oxidation and corrosion, while possessing a low density, thus making it a popular choice for aerospace applications [1]. The benefits of additive manufacturing (AM) include enabling the production of geometrically complex parts, decreasing the amount of scrap material during part production, and reducing the time associated with routing through the supply chain needed to produce parts, and as such, Ti-6Al-4V (Ti64) has been a popular choice for various means of AM production, such as directed energy deposition, selective laser melting, and electron beam melting (EBM) [2]. In recent reviews, there has been a range of microstructures and resulting mechanical behavior (in this case, we focus on strength properties) exhibited by AM Ti64; for instance, please see [3,4,5,6,7].

**Figure 1:**
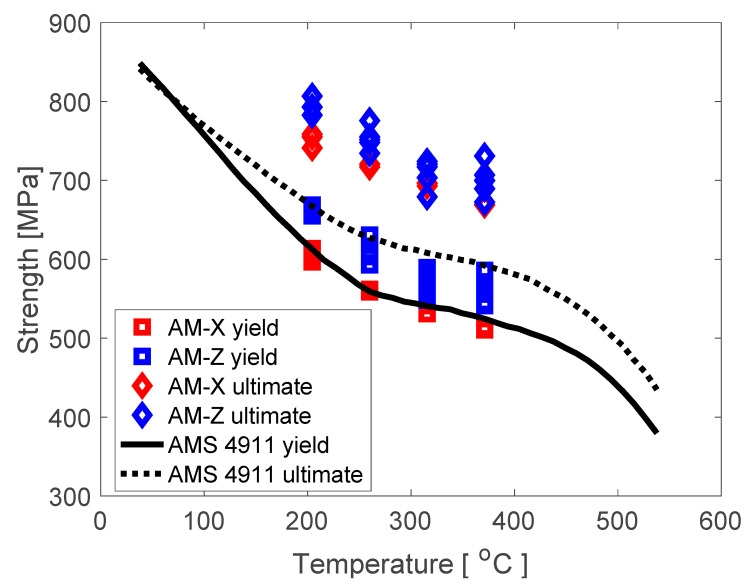
The 0.2% offset yield and ultimate strengths of additively manufacturing (AM) samples in the vertical (Z) and horizontal (X) build directions versus the baseline rolled plate (AMS 4911) as a function of test temperature.

Significant work has focused on explaining the differences in microstructures produced via AM as compared to conventional methods, in order to tailor the Ti64 microstructures to achieve optimized performance [3,4,5,6,7]. Of particular note are recent results by the authors shown in Figure 1, in which EBM Ti64 is shown to exhibit improved yield and ultimate strength properties over a range of elevated temperatures for specimens that are built with the loading axis aligned longitudinally and transverse to the AM build direction, compared to conventional AMS 4911 rolled plate (with specimens extracted along the rolling direction). While several recent studies have demonstrated that with appropriate post-processing, it is possible to achieve comparable mechanical behavior to conventional wrought Ti64, it is unexpected to produce enhanced strength properties, on average, across a statistically representative set of specimens. Thus, the focus of the present study is to ascertain the potential rationale for the enhanced strength exhibited by the EBM Ti64 material.

Ti-6Al-4V microstructures produced via AM techniques exhibit distinct characteristics, in their as-built conditions, compared to conventional processing. First, due to the rapid cooling rates, a martensitic, α’, microstructure is initially observed [8,9]. During the build process, a lower energy density will result in finer α lathes [10,11]. Further, due to the relatively high strength of the pyramidal slip systems, typical Ti-6Al-4V microstructures possess a relatively small dislocation density on the pyramidal slip systems, as compared to the prismatic and basal slip systems [12]. Kapoor et al. showed a high density of <c + a> dislocations for as-built AM Ti-6Al-4V microstructures [13]. Kapoor et al. explained that due to the rapid cooling, the material will experience a contraction of the crystalline structure that must be accommodated along the <c> axis of the material, thus generating <c + a> dislocation structures, which are less common in conventional materials [13]. Further, the steep thermal gradients during AM processing result in the growth of columnar β grains, which has been widely documented [8,14,15,16,17,18,19,20,21]. A strong fiber texture exists within the epitaxial β grains orientated in the <001> directions parallel to the build [22]. Experimental techniques have used digital image correlation to investigate heterogeneous deformation and strain accumulation relative to the microstructural features in additively manufactured materials, including Ti64 [9,13] and other material systems [23,24,25].

In this study, the role of the columnar prior β grains and fiber texture indicative of the AM processing are systematically probed in EBM Ti-6Al-4V. Specifically, the grain structure is characterized in various build orientations and compared to conventional Ti-6Al-4V from the rolled plate (AMS 4911). The grain orientations and morphologies of the prior β grains are reconstructed via the Burgers orientation relationship. Crystal plasticity simulations are performed on the α microstructures, as well as the prior β microstructures. The results are used to rationalize the role of the prior β fiber texture on the strength characteristics of this material.

## 2. Materials and Manufacturing

### 2.1. Additive Manufacturing and Prior Studies

**Figure 2:**
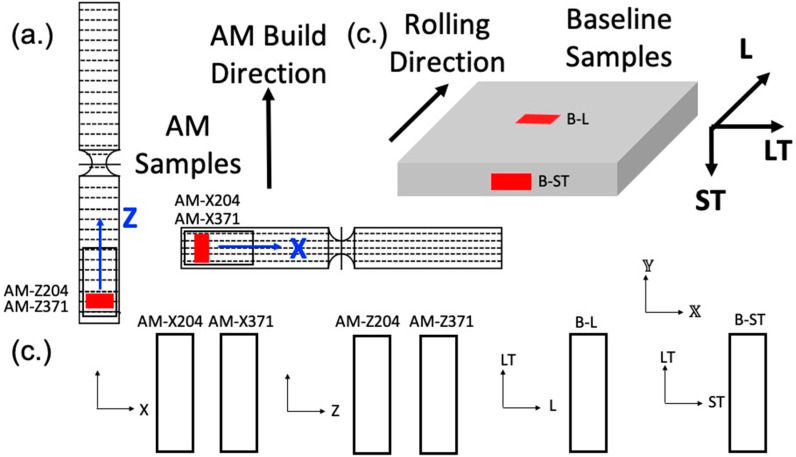
(**a**) Vertically built (orientated with respect to the Z axis: AM-Z204 and AM-Z371) and horizontally (orientated with respect to the X axis: AM-X204 and AM-X371) built AM samples, with the red indicating the location of microstructure characterization. (**b**) The baseline rolled plate for longitudinal (B-L) and transverse (B-ST) orientated samples with red indicating the location of microstructure characterization. (**c**) Orientations of each of the samples that underwent microstructure characterization. For subsequent elasto-viscoplastic (EVP)-FFT modeling, the samples were each loaded along the 𝕏 direction.

Test samples were produced in an ARCAM A2X using the recommended process parameters. In an EBM process, the beam power and scan speed are not explicitly controlled, but rather are implicitly controlled via a machine speed function to achieve a targeted melt pool size. The maximum current was set to 17 mA; the line offset was 0.2 mm; the focus offset was 10 mA; and the speed function index was 36. A complete set of process parameters, including preheat (which reduces residual stresses and potentially limits epitaxial grain growth) and contour settings, were described in [26]. Further, the powder size distribution and composition, along with porosity present in the powder and the build, have been shown by a previous study [26]. Within the powder bed, the vertical axis (aligned with the build direction) was Z, the powder raking axis X, and the cross-product Y. Standard dog bone specimens were built with the tensile axis aligned parallel to the build direction (referred to as Z orientation or vertically built) and orthogonal to the build direction (referred to as X orientation or horizontally built), as shown in Figure 2. Following the build process, these samples underwent a hot isostatic pressing (HIP) operation at 1650 +/− 14 °C for 2 h at 103.4 +/− 3.4 MPa.

A total of 8 specimens of Ti-6Al-4V material produced via AM, specifically powder bed, electron beam melted, and a total of 2 baseline specimens of Ti-6Al-4V material from a rolled plate (AMS 4911) were characterized. The 8 HIPed EBM-built Ti64 specimens were obtained from the grip of tensile coupons, where 4 coupons had the X axis of the build aligned with the tensile axis of the coupon (horizontally built) and 4 coupons had the Z axis of the build aligned with the tensile axis of the coupon (vertically built). Each set of 4 coupons was subjected to the following temperatures during elevated temperature tensile testing: 204 °C, 260 °C, 316 °C, and 371 °C, respectively. The tensile tests were performed by Northrop Grumman Corporation following ASTM E21 standards [27]. To simplify the analysis of EBM Ti64 at high temperatures, only the lower and upper temperature bounds were analyzed, that is only the specimens at 204 °C and 371 °C were analyzed, as the grain structure was not expected to change (as samples were extracted from the grip region of each specimen). Therefore, a total of 6 samples were characterized: 2 X axis AM specimens (horizontally built) extracted from the grip regions of tensile coupons tested at 204 °C and 371 °C, 2 Z axis AM specimens (vertically built) extracted from the grip regions of tensile coupons tested at 204 °C and 371 °C, and 2 baseline samples from (AMS 4911) the rolled plate along the longitudinal and transverse directions.

### 2.2. Microstructure Characterization

The grain structure and orientation information were captured for each of the six specimens via electron backscatter diffraction (EBSD). First, each sample was prepared for the EBSD characterization. To analyze the equivalent orientation on the rolled plate, the first baseline specimen was analyzed along the L-LT plane (aligned with the rolling direction) analogous to the coupons manufactured along the Z axis (vertically built), and the second baseline specimen was analyzed along the ST-LT plane (transverse to the rolling direction) analogous to the coupons manufactured along the X axis (horizontally built). A summary of all 6 specimens studied can be seen in Table 1.

**Figure 3:**
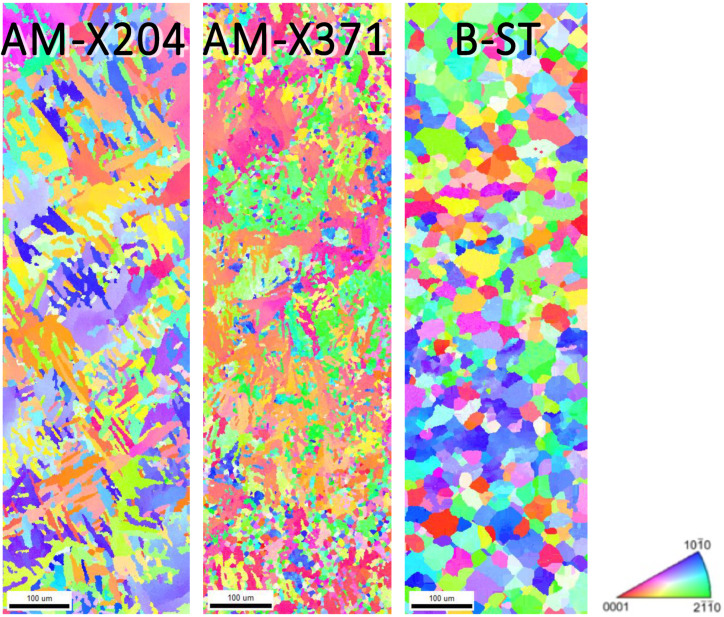
Electron backscatter diffraction (EBSD) scans of the α phase for the X oriented (horizonal built) AM specimens at 204 °C and 371 °C and the analogous baseline ST-LT plane (transverse to the rolling direction). The scale marker shown is 100 μm in length.

**Figure 4:**
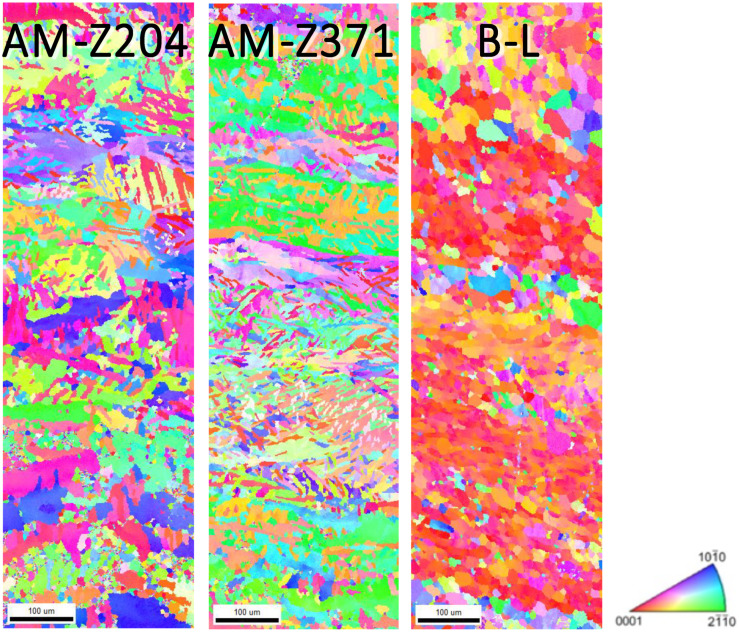
EBSD scans of the α phase for the Z oriented (vertically built) AM specimens at 204 °C and 371 °C and the analogous baseline L-LT plane (aligned with the rolling direction). The scale marker shown is 100 μm in length.

All specimens were mounted in Bakelite and sequentially polished in the final step using a 1200 grit SiC sand paper and distilled water, followed by both rotary and vibratory polishing with a solution containing four parts 0.05 μm colloidal silica and one part 30% hydrogen peroxide, to obtain the mirror-like surface necessary for EBSD analysis. Prior to EBSD, each specimen was cleaned via both rinsing and sonicating using distilled water, isopropyl alcohol, acetone, and methanol, in that order. Electron backscatter diffraction characterization was conducted using a Quanta 650 FEG scanning electron microscope with an EDAX EBSD camera and the TEAM Collection and Analysis software. The SEM setup parameters were as follows: a 20 kV accelerating voltage, a spot size of 4, a 100 μm aperture, a working distance of 15–20 mm, a 250× magnification, and a 70° tilt. The EDAX EBSD camera parameters were as follows: a gain of 507, 11.35 ms of exposure time, 4 × 4 binning, a snapshot average of 5, and a step size of 0.7 μm. The area characterized on each specimen was a 300 μm × 1000 μm region across the diameter of the grip, as seen in Figure 2. The average duration of each EBSD scan was ~3 h. Given the relatively large step size, only the α phase was characterized for each EBSD scan. Figure 3 and Figure 4 displays the EBSD characterization for the transversely and longitudinally oriented specimens, respectively.

### 2.3. Identification of the Prior β Microstructural Features

A crystallographic relationship exists between the prior β phase that forms during the high temperature electron beam melting process and the α phase present at room temperature. Using this relationship, the morphology and orientation of the prior β phase (with the BCC crystal structure) can be unambiguously reconstructed from the characterization of the α phase (with the HCP crystal structure). The Burgers orientation relationship (BOR) provides a crystallographic equivalency and alignment between the α and β phases [28], such that:

(1) $${\left(0001\right)}_{\alpha }//{\left\{101\right\}}_{\beta }$$

(2) $$11\bar{2}0_{\alpha }//{\left\{111\right\}}_{\beta }$$

**Figure 5:**
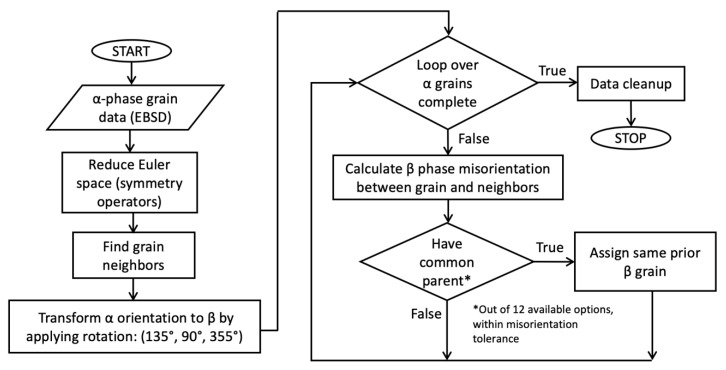
Flowchart describing the algorithm used to calculate the prior β phase using α phase EBSD data.

For each orientation of the β phase, twelve possible crystallographic variants for the α phase exist, due to crystallographic symmetries. From the EBSD characterization of the α phase, the BOR was used via an in-house MATLAB script to automate the reconstruction of the prior β phase, based on the procedure described by Humbert et al. [29]. The process used to obtain the prior β grain morphology and orientation was as follows. First, a set of symmetry operators was applied to the α grains to reduce their representation in Euler space. From the BOR, the conversion between the α and β phase can be represented using a rotation matrix with Bunge Euler angles (135°, 90°, 355°) [30]. All possibilities of the prior β grain orientation could be determined for each α grain by applying the rotation matrix and crystal symmetries. The neighbors for each α grain were determined, and the conversion between each α grain and all possibilities of the prior β phase were calculated. If the α grain in consideration and its neighbor had a common parent β grain (from all possible parent β variants), both grains were assigned as being part of the same parent β grain. For this equivalency between neighboring grains, a misorientation threshold of 5° was applied. The algorithm investigated all the neighboring grains for each grain in consideration, until each was assigned to a parent β orientation. In case multiple variants could be assigned to a set of α grains, the variant with the least misorientation was chosen. The entire process consisted of the algorithms looping over each neighboring grain until the α EBSD grain maps were converted into grain maps of the prior β phase, and in the process determining the grain morphology and orientation of the prior β parent grains. The algorithm used to recreate the prior β phase is described using a flowchart in Figure 5.

**Figure 6:**
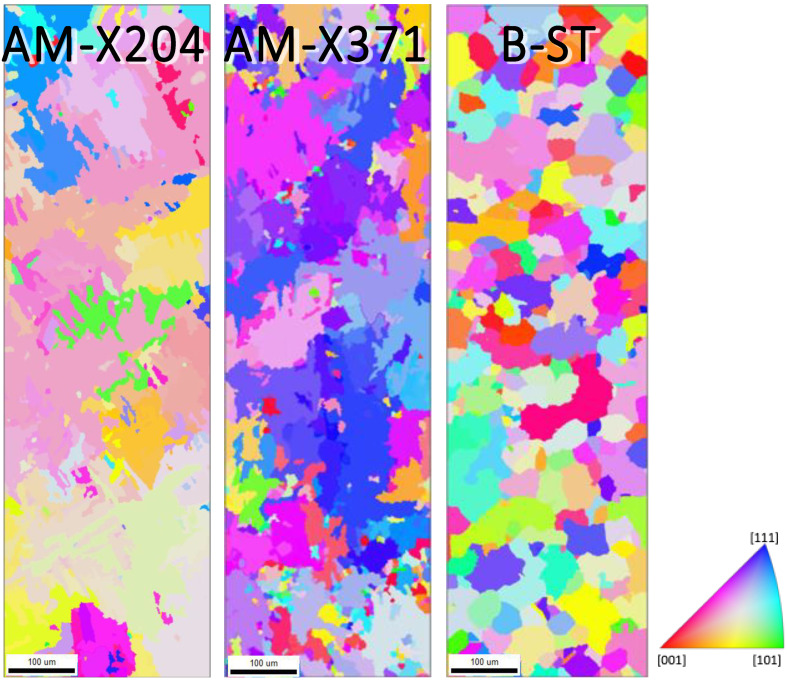
Prior β reconstructions for the X oriented (horizonal built) AM specimens at 204 °C and 371 °C and the analogous baseline ST-LT plane (transverse to the rolling direction). The scale marker shown is 100 μm in length.

**Figure 7:**
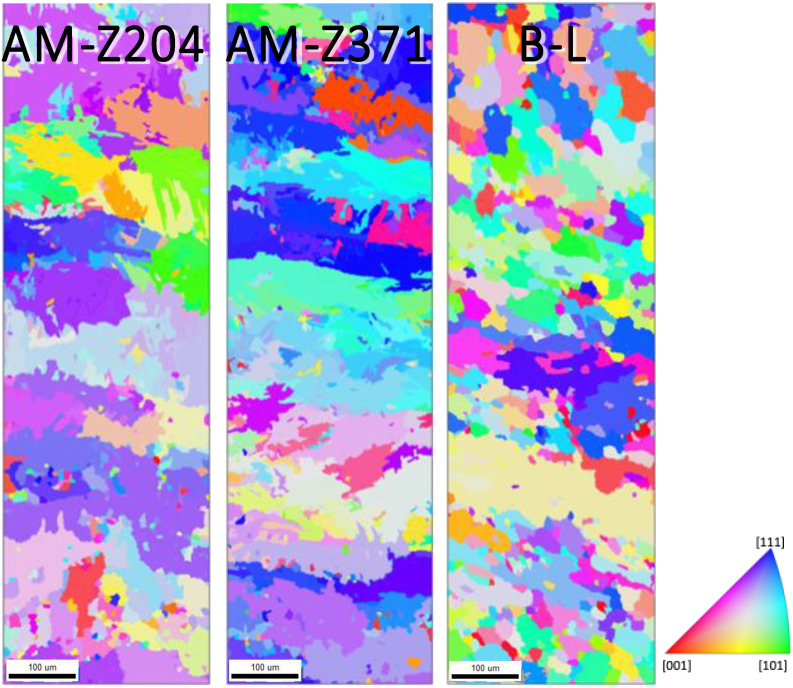
Prior β reconstructions for the Z oriented (vertically built) AM specimens at 204 °C and 371 °C and the analogous baseline L-LT plane (aligned with the rolling direction). The scale marker shown is 100 μm in length.

As previously mentioned, epitaxial grain growth of the β phase occurs during the build process aligned with the <001>. To understand the morphological ramifications that this textured structure has on the strength properties, the α microstructure was transformed into its prior β phase. The microstructure of the equivalent prior β for both the transversely and longitudinally oriented AM specimens, as well as their rolled plate equivalents can be seen in Figure 6 and Figure 7, respectively.

## 3. Crystal Plasticity Modeling Framework

To determine the strength attributes for each of the six microstructures, the grain maps were instantiated for use within an elasto-viscoplastic fast Fourier transform (EVP-FFT) representation of a crystal plasticity model. Additional details of the EVP-FFT formulation were described by Lebensohn et al. [31], based on the original formulation by Moulinec and Suquet [32]. The EVP-FFT formulation allows the imposition of a macroscopic strain rate, a macroscopic stress, or a combination of both. Here, a parallelized implementation of the EVP-FFT model was used [33]. For this model, only a macroscopic strain rate, $\dot{E}$, along the X-direction (as indicated in Figure 2c) was imposed, while the other strain components were adjusted to fulfill stress-free conditions. To comply with the 2^n^ periodic requirement of the Fourier method, all microstructures were padded with dummy gas phases (on the Y faces of the microstructure as indicated in Figure 2c), with the microstructure being padded with a solid phase on the X faces to ensure axial loading in the material. Each microstructure was subjected to 1% applied loading in the X-direction.

The EVP-FFT formulation calculates a compatible strain field and an equilibrated stress field that satisfy the constitutive relation at every domain point, x. Hooke’s law is expressed, in the crystal frame, at time t+Δt:

(3) $$\sigma \left(x\right)=C\left(x\right):\varepsilon^{el}\left(x\right)=C\left(x\right):\left(\varepsilon \left(x\right)-\varepsilon^{pl}\left(x\right)\right)=C\left(x\right):\left(\varepsilon \left(x\right)-\varepsilon^{pl,t}\left(x\right)-{\dot{\varepsilon }}^{pl}\left(x\right)\Delta t\right)$$

where C(x) is the fourth-order stiffness tensor and $\varepsilon^{pl,t}$ is the plastic strain at time t, and the plastic strain rate is described as:

(4) $${\dot{\varepsilon }}^{pl}\left(x\right)=\overset{N}{\underset{\alpha =1}{\sum }}M^{\alpha }\left(x\right){\dot{\gamma }}^{\alpha }\left(x\right)={\dot{\gamma }}_{0}\overset{N}{\underset{\alpha =1}{\sum }}M^{\alpha }\left(x\right){\left(\frac{\left|M^{\alpha }\left(x\right):\sigma \left(x\right)\right|}{\bar{\tau }\left(\Gamma ,\;x\right)}\right)}^{n}sgn\left(M^{\alpha }\left(x\right):\sigma \left(x\right)\right)$$

where N is the number of possible slip systems, ${\dot{\gamma }}^{\alpha }$ is the resolved shear strain rate for slip system α, $\bar{\tau }\left(\Gamma ,x\right)$ is the critical resolved shear stress (CRSS) for a material point, x, which is dependent on the accumulated strain, Γ, experienced at that point, and $M^{\alpha }\left(x\right)$ is the symmetric Schmid tensor at a spatial point, x, for slip system α. Moreover, ${\dot{\varepsilon }}^{pl}\left(x\right)$ and σ(x) are the plastic strain rate and stress tensor at each spatial point, x. The flow rule has two additional material parameters, where ${\dot{\gamma }}_{0}$ is the reference shear strain rate and n is the rate sensitivity exponent. This phenomenological relationship expresses the rate of crystallographic slip, mediated by dislocation motion, on an individual slip system, based on the resolved shear stress on the same slip system, $\tau^{\alpha }$, which is related to the stress tensor σ(x) as follows:

(5) $$\tau^{\alpha }\left(x\right)=M^{\alpha }\left(x\right):\sigma \left(x\right)=\frac{1}{2}\left(m^{\alpha }\left(x\right)⨂n^{\alpha }\left(x\right)+n^{\alpha }\left(x\right)⨂m^{\alpha }\left(x\right)\right):\sigma \left(x\right)$$

where $n^{\alpha }$ is the normal of the slip plane and $m^{\alpha }$ is the slip direction. As a slip system experiences dislocation motion resulting in crystallographic slip, the elastic strain fields produced via the dislocation mechanics result in the hardening of the slip system. For the present case, this phenomenological behavior was modeled using a generalized Voce hardening law, which was chosen as it appropriately captured the mechanical behavior of the material, while introducing a minimum number of model parameters, thus reducing the overall uncertainty of the model. The generalized Voce hardening law evolves the critical resolved shear stress at a particular spatial point, x, as a consequence of the accumulated shear strain across all slip systems for that point:

(6) $$\bar{\tau }\left(\Gamma ,x\right)=\tau_{o}+\left(\tau_{1}+\theta_{1}\Gamma \left(x\right)\right)\left[1-e^{-\frac{\Gamma \left(x\right)\theta_{0}}{\tau_{1}}}\right]$$

where $\tau_{0}$ and $\theta_{0}$ are the initial yield stress and hardening rate, respectively, and $\tau_{1}$ and $\theta_{1}$ are the parameters that describe the asymptotic behavior of the material [34].

**Figure 8:**
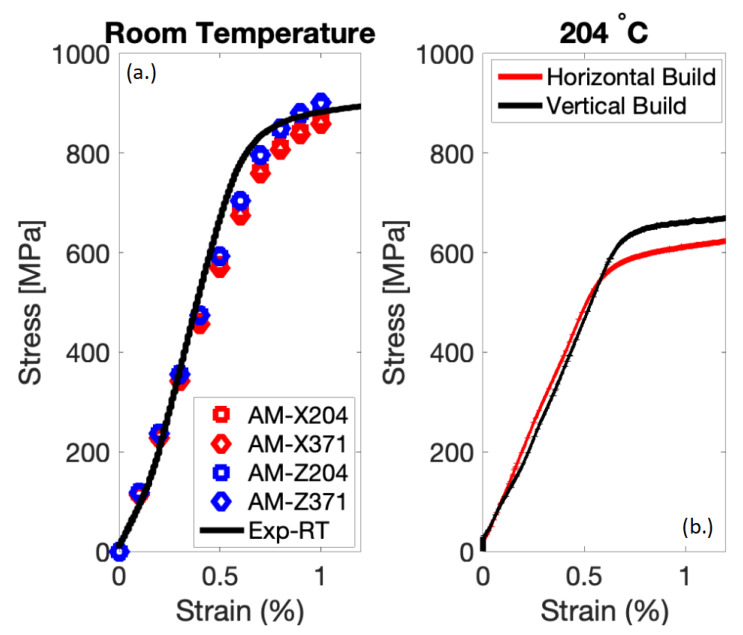
(**a**) At room temperature, stress-strain curves from the EVP-FFT simulation for the α phases of the horizontally built (X) and vertically built (Z) samples compared to an experiment for the vertically built sample. (**b**) At 204 °C, experimental results comparing the stress-strain response for horizontally built (X) and vertically built (Z) samples.

The corresponding model parameters were calibrated against test data at room temperature, in order to obtain a comparative assessment of the strength characteristics of the AM and baseline materials for the longitudinal and transverse orientations. As expected, the slip characteristics and kinetics were expected to change with increasing temperature, although this is beyond the scope of the present paper. For the α phase, the parameters corresponding to the elastic regime, i.e., the elastic constants, were taken from the work of Bridier et al. [35]. The plastic parameters, corresponding to the Voce hardening law, were obtained by manually fitting to macroscopic stress-strain curves. Since the Voce-type hardening law is an empirical relationship, obtaining parameters by fitting to macroscopic stress-strain curves was a suitable option. To ensure that the fitting procedure resulted in physically relevant parameters, the ratio of the initial CRSS values of the α (HCP) slip systems (prismatic, basal, and pyramidal) were taken from the literature [35,36,37] and are as follows: τ_pyramidal_/τ_basal_ = 1.40 and τ_prism_/τ_basal_ = 0.88. After fixing the elastic constants and the ratio of the CRSS values of the α (HCP) slip systems, the plastic parameters for the EVP-FFT model were fit to macroscopic stress-strain curves, obtained from specimen-level tensile tests (by ensuring that the simulated stress-strain curves matched the ones obtained from experiments, as shown in Figure 8a). It should be noted that the material behavior was simulated at room temperature, whereas only the input microstructure taken from the six specimens in Table 1 was varied to account for grain morphology and texture variations due to the AM processing. For comparison, the experimental stress-strain data are shown for two build directions in Figure 8b at 204 °C; however, all results that follow were simulated for room temperature. For the simulations of the reconstructed β phase, the elastic constants were taken from the work of Bernier et al. [38]. Since the β phase is mechanically softer than the α phase, in previous studies, the plastic properties (the initial CRSS value and the associated hardening) of the β phase slip systems were taken to be the same as [39] or slightly lower [37] than that of the α prismatic slip system. In this work, the plasticity parameters of the β phase were taken to be the same as that of the α prismatic slip system (which corresponded to the softest α slip system) to reflect the current literature, as done by Kasemer et al. [39]. The final set of plastic and elastic parameters used in the work is given in Table 2 and Table 3, respectively. It was expected that the crystal plasticity parameters would be different for the AM and plate material, based on the substructure and higher pyramidal dislocation density in the AM material [13]. However, as a first order approximation, all material parameters were kept constant for the AM and plate Ti64 material to focus on the difference of the grain morphology and texture on strength predictions.

## 4. Results

**Figure 9:**
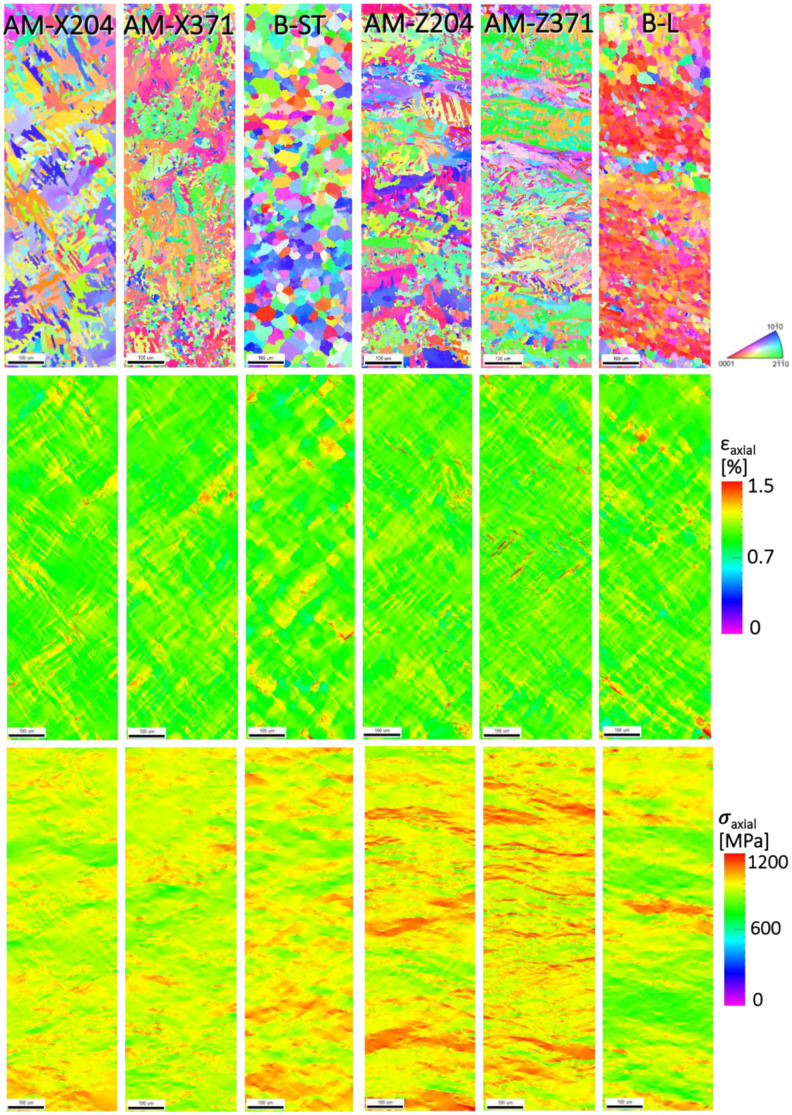
For the prior α microstructures (top row) in all six specimens, the strain (middle row) and stress (bottom row) distributions aligned with the axial loading direction are shown, from the EVP-FFT simulation after being loaded to 1% strain. The scale marker shown is 100 μm in length.

For the EVP-FFT micromechanical models of the α phase (microstructures shown in Figure 3 and Figure 4), the resulting strain and stress distributions are shown in Figure 9. The axial component of the total strain and stress tensors is plotted, that is, the XX component, the component aligned with the loading direction of the simulation. The strain distributions for the α phase remained qualitatively similar across each of the different specimens, with wider areas containing higher strains for the B-ST and B-L microstructures. In contrast, the stresses showed a markedly different behavior between the longitudinally and transversely oriented microstructures. The transverse microstructures (aligned with the X direction of the build, horizontally built) displayed lower stresses within the grains, with a higher stress being observed in their baseline rolled plate counterpart, the B-ST microstructure. The longitudinal microstructures (aligned with the Z direction of the build, vertically built) exhibited higher stresses and regions of high stress with patterns aligned with the AM build direction. Similarly, the counterpart B-L microstructure displayed stress patterning that was oriented along the rolling direction; however, there were fewer areas experiencing high stress levels. The average stress-strain response for the microstructure showed that, in general, the AM material had higher strength characteristics for the longitudinal microstructures compared to the baseline B-L microstructure, whereas there were no appreciable differences in the strength characteristics between the transverse microstructures (AM samples built in the horizontal direction and baseline B-ST specimen).

**Figure 10:**
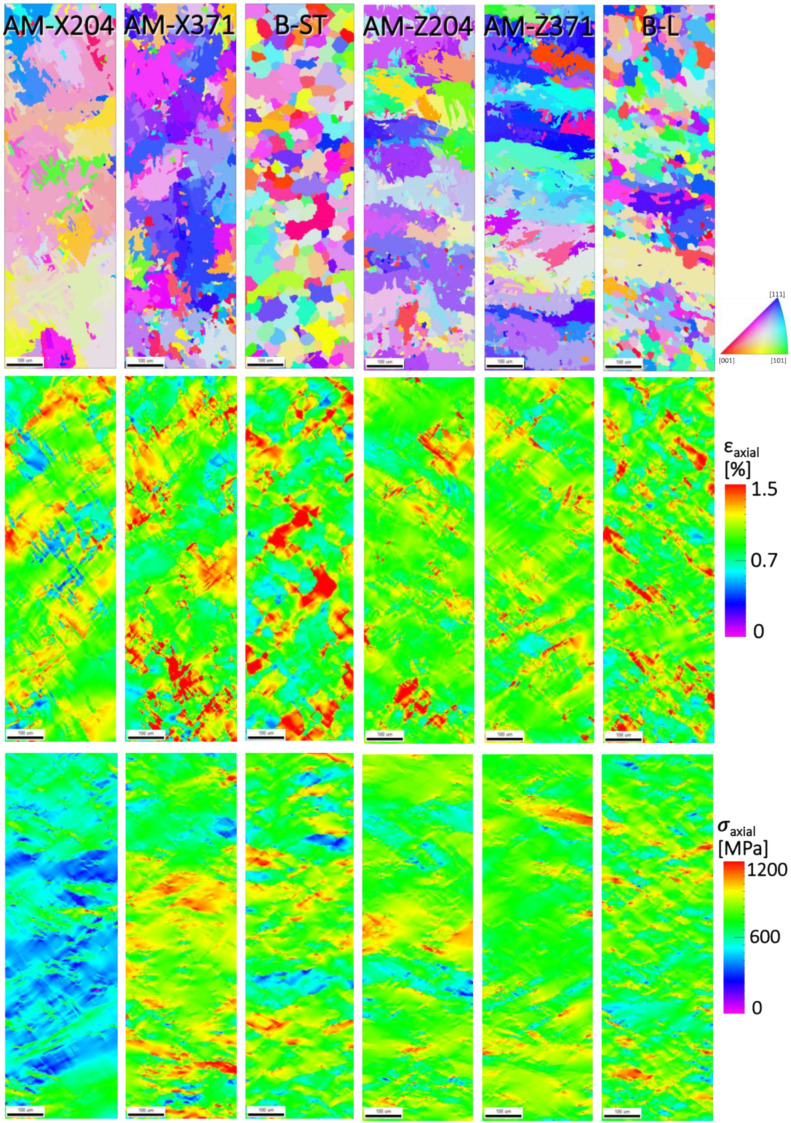
For the prior β reconstructed microstructures (top row) in all six specimens, the strain (middle row) and stress (bottom row) distributions aligned with the axial loading direction are shown, from the EVP-FFT simulation after loading to 1% strain. The scale marker shown is 100 μm in length.

As previously mentioned in Section 2.3, the prior β grain structure was reconstructed from the EBSD characterization of the α phase. Each of the prior β grain structures were used within the EVP-FFT micromechanical models (Figure 6 and Figure 7), and the resulting axial components of the total strain and stress tensors are shown in Figure 10. As shown in Figure 10, the axial strain distributions for the prior β phases of the transverse specimens (horizontally built directions) exhibited a higher level of strain localizations than the longitudinal specimens (vertical build directions). The strain fields were localized at the prior β boundaries, in agreement with the observations by Book and Sangid [9], in which the prior β boundaries (and the corresponding changes in orientation of the associated crystallographic slip systems) resulted in resistance to slip transmission. When comparing the AM specimens with the baseline Ti64 specimens, higher strain localizations could be observed for the equivalent baseline B-ST and B-L microstructures compared to the associated AM microstructures. On the other hand, Figure 10 shows the stress distributions for the prior β reconstructed microstructures, with most of the microstructural maps of the stress fields exhibiting qualitatively similar behavior, with the exception of AM-X204 (microstructure of the AM horizontally built specimen, tested at 204 °C). When analyzing this microstructure (see Figure 6), it could be observed that the AM horizontally built microstructure displayed a heavily textured prior β reconstructed microstructure, with the majority of the grains exhibiting a soft orientation, thus affecting the overall stress distribution of the simulations shown. By viewing the average response of the simulated prior β polycrystalline aggregate, it could be observed that, in general, the AM material had a higher strength response for microstructures from both build directions compared to the baseline materials from the corresponding oriented rolled plate, with the one exception of the prior β reconstruction derived from the horizontally built microstructure tested at 204 °C (AM-X204), most likely due to the soft microtextured region.

## 5. Discussion

**Figure 11:**
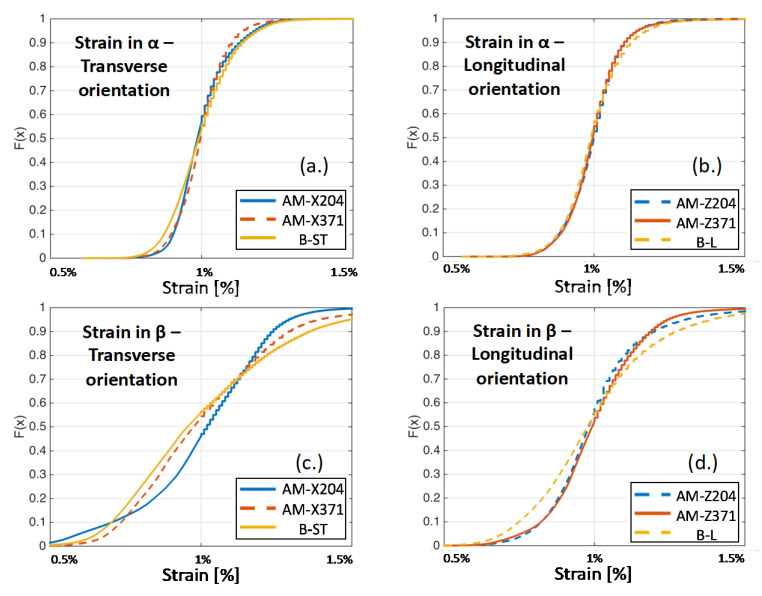
CDF plots of the strain distributions aligned with the axial loading direction for all specimens: (**a**) α grains in the transversely oriented samples, (**b**) α grains in the longitudinally oriented samples, (**c**) prior β grains in the transversely oriented samples, and (**d**) prior β grains in the longitudinally oriented samples.

**Figure 12:**
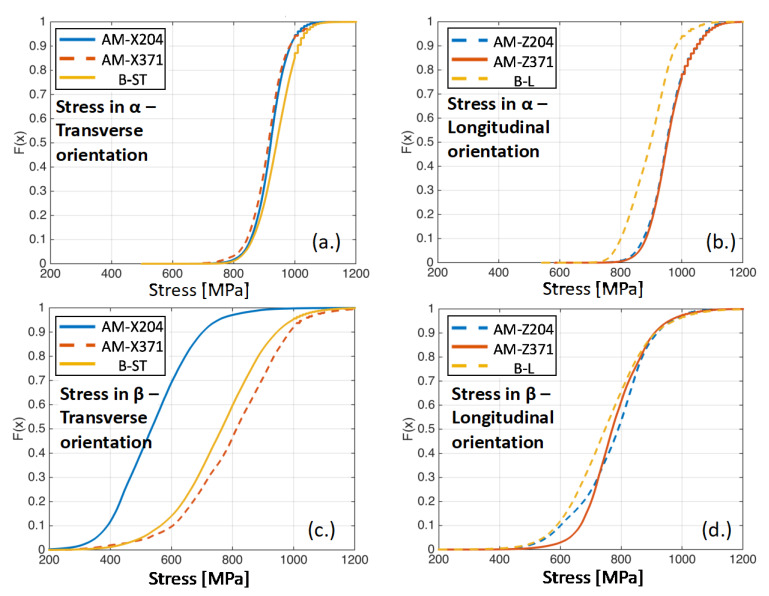
CDF plots of the stress distributions aligned with the axial loading direction for all specimens: (**a**) α grains in the transversely oriented samples, (**b**) α grains in the longitudinally oriented samples, (**c**) prior β grains in the transversely oriented samples, and (**d**) prior β grains in the longitudinally oriented samples.

In order to analyze the heterogeneity in the micromechanical fields for each of the simulated microstructures, the cumulative distribution function (CDF) plots were constructed, shown as F(x). The strains for the α microstructures in the transverse and longitudinal orientations are shown in Figure 11a,b, respectively, and the prior β reconstructed microstructures for the transverse and longitudinal orientations are shown in Figure 11c,d, respectively. Likewise, the CDF plots for the same microstructures are shown for the stress distributions in Figure 12. From Figure 11, more than 95% of the strain values (bound between zero and 0.95 on the CDF plot) were between 0.5% and 1.5% strain for the simulations, which were subjected to 1% nominal strain. There was no significant difference in the strain distributions between the AM and baseline rolled microstructures for the α phase simulations. On the other hand, there was a significant difference in the strain distributions derived from the β phase reconstructions, which elucidated the role of the <001> textured prior β phase in the AM materials for regions of high strain localization in the microstructure.

Figure 12 shows that, for the α phase, the stresses exhibited within the simulations were slightly higher for the baseline B-ST microstructure compared to the AM material in the transverse orientations (corresponding to the horizontal build direction). On the contrary, the stresses from the simulations were lower for the baseline B-L microstructure than for the AM material in the longitudinal orientations (corresponding to the vertical build direction). This asymmetry in stress distributions in the α phase of the AM microstructures for the various orientations compared to their conjugate baseline samples highlighted the texture development (primarily the hard/soft orientations of the HCP phase for the vertical/horizontal build directions) during the build process. For both orientations, there was no appreciable difference in strain distributions for the simulations of the different AM samples; recall the boundary conditions for each simulation subjected the microstructure to a prescribed velocity field and were, in essence, strain controlled. For the simulations of the prior β phase reconstructions, the baseline B-ST microstructure had slightly lower stresses than the transversely orientated (horizontally built) microstructure extracted from the sample tested at 371 °C and significantly higher stresses than the microstructure extracted from the sample tested at 204 °C. It should be noted that the lower stresses for the horizontally built AM material were due to heavy texturing in the relatively small characterized region, as discussed earlier. On the other hand, the stress distributions in the prior β phase reconstructions were lower for the baseline B-L microstructure than for the AM longitudinally orientated (vertically built) microstructures.

In general, the baseline Ti64 material appeared to have regions of higher accumulated strains than the AM material for most of the cases analyzed, whereas the stresses seemed to vary depending on the build orientation and the phase analyzed: the stresses for the baseline material were lower for both simulations of the α and β phases for the longitudinal orientations (compared to the vertically built AM samples), slightly higher for the transverse orientations (horizontally built AM microstructures) for the α phase, and in between the stresses for the AM materials of the transverse orientations (horizontally built AM microstructures) for the prior β phase.

## 6. Conclusions

In this study, the microstructure of Ti-6Al-4V produced via EBM was evaluated via EBSD characterization and compared to the associated orientations of the baseline rolled plate. Crystal plasticity simulations were employed to identify strain localization and stress concentration relative to the microstructural features. The conclusions from this work are as follows:

1. The AM material exhibited epitaxial growth of the β grains due to a steep thermal gradient during processing. Using the procedure outlined by Humbert et al. [29], the prior β phases were reconstructed and shown to exhibit a strong fiber texture and columnar grain morphology aligned with the build direction.
2. The EBM Ti-6Al-4V exhibited improved strength properties for specimens with their axis aligned along and transverse to the build direction compared to specimens from the baseline rolled plate along and transverse to the rolling direction, respectively.
3. The micromechanical fields for the crystal plasticity simulations with the α microstructure were qualitatively similar for the AM and baseline materials, while, in general, the AM material exhibited less regions of intense strain localization and higher stresses for the simulations on the prior β grain microstructures.
4. Therefore, it could be seen for the longitudinal and transverse microstructures that the columnar and fiber textured prior β grain structure in AM material could significantly influence the mechanical behavior of the material and should be taken into account when considering the performance of AM Ti-6Al-4V in the as-built condition.

## Figures and Tables

**Table 1:** Summary of the six specimens analyzed in this study, including orientation, processing route, and naming convention. EBM, and electron beam melting.

Orientation	Processing Route	Description	Naming Convention
Longitudinal	EBM AM	Vertically built specimen tested at 204 °C	AM-Z204
		Vertically built specimen tested at 371 °C	AM-Z371
	Rolled Plate	Baseline sample from the L-LT plane	B-L
Transverse	EBM AM	Horizontally built specimen tested at 204 °C	AM-X204
		Horizontally built specimen tested at 371 °C	AM-X371
	Rolled Plate	Baseline sample from the ST-LT plane	B-ST

**Table 2:** Crystal plasticity parameters used in the EVP-FFT model at room temperature.

	α (HCP)			β (BCC)
Slip System Family	Prismatic	Basal	Pyramidal	(110)<111>
$\tau_{o}$ (MPa)	199	226	317	199
$\tau_{1}$ (MPa)	58	66	93	58
$\theta_{0}$ (MPa)	6000	6000	6000	6000
$\theta_{1}$ (MPa)	16	18	25	16
Number of slip systems	3	3	12	12
n	15			
${\dot{\gamma }}_{o}$	0.001			

**Table 3:** Elastic coefficients used in the EVP-FFT model at room temperature. All values in GPa.

	$C_{11}$	$C_{22}$	$C_{33}$	$C_{12}$	$C_{13}$	$C_{23}$	$C_{44}$	$C_{55}$	$C_{66}$
α	162.4	162.4	180.7	92	69	69	49.7	49.7	35.2
β	135	135	135	113	113	113	54.9	54.9	54.9
